# Extracellular Vesicles from Adipose Tissue Stem Cells in Diabetes and Associated Cardiovascular Disease; Pathobiological Impact and Therapeutic Potential

**DOI:** 10.3390/ijms21249598

**Published:** 2020-12-16

**Authors:** Alina Constantin, Alexandru Filippi, Nicoleta Alexandru, Miruna Nemecz, Adriana Georgescu

**Affiliations:** Institute of Cellular Biology and Pathology “Nicolae Simionescu” of Romanian Academy, 050568 Bucharest, Romania; alina.constantin@icbp.ro (A.C.); alexandru.filippi@icbp.ro (A.F.); nicoleta.alexandru@icbp.ro (N.A.); miruna.nemecz@icbp.ro (M.N.)

**Keywords:** adipose tissue-derived stem cells, extracellular vesicles, diabetes, cardiovascular disease

## Abstract

Adipose tissue-derived stem cells (ADSCs) are pluripotent mesenchymal stem cells found in relatively high percentages in the adipose tissue and able to self-renew and differentiate into many different types of cells. “Extracellular vesicles (EVs), small membrane vesicular structures released during cell activation, senescence, or apoptosis, act as mediators for long distance communication between cells, transferring their specific bioactive molecules into host target cells”. There is a general consensus on how to define and isolate ADSCs, however, multiple separation and characterization protocols are being used in the present which complicate the results’ integration in a single theory on ADSCs’ and their derived factors’ way of action. Metabolic syndrome and type 2 diabetes mellitus (T2DM) are mainly caused by abnormal adipose tissue size, distribution and metabolism and so ADSCs and their secretory factors such as EVs are currently investigated as therapeutics in these diseases. Moreover, due to their relatively easy isolation and propagation in culture and their differentiation ability, ADSCs are being employed in preclinical studies of implantable devices or prosthetics. This review aims to provide a comprehensive summary of the current knowledge on EVs secreted from ADSCs both as diagnostic biomarkers and therapeutics in diabetes and associated cardiovascular disease, the molecular mechanisms involved, as well as on the use of ADSC differentiation potential in cardiovascular tissue repair and prostheses.

## 1. Introduction

The adipose tissue has been considered for many years an inert storage depot for nutrients, but now many evidences show that adipose tissue has various physiological roles that include regulation of metabolism, immunity and endocrine function. The anatomical location in coordination with the dynamic changes of cellular component has a prominent effect on the biology of adipose tissue. Given its central role in regulation of energy homeostasis and because obesity-related disorders such as diabetes, metabolic syndrome and cardiovascular diseases have reached an epidemic magnitude, extensive interest has been payed to establish a broad map of adipose tissue cellular composition and the intercellular communication that mediate pathologic responses.

The adipose tissue is generally divided into two distinct types, white adipose tissue (WAT) and brown adipose tissue (BAT), the first acting to store and mobilize triglycerides, and the second having the leading function of burning fatty acids and glucose for heat production, a process known as adaptive (non-shivering) thermogenesis [1].

WAT is found throughout the body but is mainly organized into anatomically distinct depots: subcutaneous WAT, which is found under the skin, and visceral WAT, which is located within the body cavities, surrounding the major organs. In addition to subcutaneous and visceral fat, WAT can be found in many other areas: breasts, on the neck and upper back, extremities, in the retro-orbital space, and within bone marrow. During the last years, a third form of adipose tissue has been characterized in rodents and humans. BAT is found as depots in newborns (in perivascular and peri-organ visceral areas) but also in adults (in cervical, supraclavicular, mediastinal, and suprarenal regions) [2]. Both visceral and subcutaneous WAT depots have been shown to harbor thermogenic adipocytes, and originally in subcutaneous WAT they were called beige, whilst in visceral WAT they were termed brite [3]. The process of browning and the recruitment of beige adipocytes typically occurs in response to certain stimuli, particularly exposure to cold or to β3—adrenergic stimulation [1,3,4]. Beige adipocytes are located within WAT depots, and are morphologically and functionally comparable to brown adipocytes [3,5]. Beige adipocytes express uncoupling protein 1 (UCP-1), a master regulator of thermogenesis in BAT [3,6].

WAT is composed of a mixed population of cells including lipid-filled mature adipocytes, and a stromal vascular fraction (SVF) that contains stromal cells (adipose stem cells, blood lineage cells, vascular cells, fibroblasts) and immune cells, the percentage of each being in a dynamic state. Healthy WAT is characterized by the presence of numerous and smaller adipocytes resulted from differentiated progenitor cells. However, it is known that WAT is highly plastic and responds to different stimuli by continuous remodeling, i.e., during periods of excess energy intake it expands by both hypertrophy (increase in cell size) and/or hyperplasia (increase in cell number) [7,8,9]. The explanation for these processes comes from the fact that within WAT there are subpopulations of adipocyte progenitor cells which exhibit different secretory pro-inflammatory or fibrotic phenotypes influencing their differentiation state. Thus, identifying different subpopulations of progenitor cells and their interaction with non-stromal resident cells in WAT it is a crucial issue in order to understand how fat homeostasis is regulated.

It has been showed that expansion of visceral WAT is mainly due to adipocyte hypertrophy that is linked to inflammation and insulin resistance [10,11]. In murine models, high fat feeding leads to adipocyte hypertrophy in both visceral and subcutaneous WAT, whereas adipogenesis is thought to occur primarily in visceral WAT [7,8]. The ability of adipose tissue to expand by hyperplasia confers a protective advantage against insulin sensitivity and metabolic disease risk by the recruitment of adipocyte progenitor cells and commitment to the adipose lineage [10,12]. Chronic overnutrition forces adipocytes to enlarge, to store more triglycerides and when their buffering capacity is exceeded, hypertrophied adipocyte death coupled with the accumulation of pro-inflammatory macrophages and fibrotic cells into the adipose tissue leads to ectopic fat accumulation in muscle and liver and to systemic insulin resistance [13,14].

Differences between visceral and subcutaneous adipose tissue exist also in terms of the number of isolated adipose tissue-derived stem cells (ADSCs) and their ability to expand in vitro. SFV cell number isolated from omental adipose tissue was significantly higher than that from subcutaneous adipose tissue in human donors [15]. In addition, it was noted that the yield of ADSCs isolated from each gram of visceral fat was significantly greater than that from subcutaneous fat, implying that the visceral adipose tissue contained more ADSCs [16].

Extracellular vesicles (EVs) are different types of submicron vesicles derived from nearly all cells in response to cell activation, stress or apoptosis. Based on the size, morphology, and mechanism of biogenesis, they are divided in exosomes and ectosomes. Exosomes (50–100 nm) are small vesicles exocyted from multivesicular bodies (MVBs) after receptor-mediated endocytosis. Ectosomes (microvesicles (MVs) or microparticls (MPs)) are slightly larger vesicles (100–500 nm) compared with exosomes and are also cell specific as they are released from plasma membrane by budding [17].

Metabolic syndrome is a multifactorial disease and involves numerous cell types, tissues, organs and humoral factors (cytokines, growth factors and miRNA molecules, many of them being encapsulated in circulating EVs). Almost any type of cells (endothelial cells, lymphocytes, T cells, macrophages, renal cells, cancer cells and stem cells) can release EVs in physiological state, and number of these submicron vesicular membrane structures is increased during pathological processes. EVs can exhibit characteristics (RNAs, DNA and lipids) of their parent cell of origin and may provide diagnostic and prognostic value in metabolic dysfunction and cardiovascular diseases [18,19,20]. EVs can be isolated from plasma, urine, cerebral spinal fluid, lymph and conditioned media from cell culture by different methods.

Mesenchymal stem cells (MSCs) are multipotent cells with high proliferative, self-renewal, multi-lineage differentiation, and regenerative potential. MSCs reside in variable amount in adult organs of human bodies with high regeneration and differentiation capacity, but bone marrow, human umbilical cord and adipose tissue are important origins of MSCs for researches. MSCs from these sources exhibit common features in terms of proliferation and differentiation capacity, but their regenerative potential is site-specific. Previous studies [21,22] showed that MSCs convey their reparative effects by releasing EVs, including small and large EVs. Recently, MSC-derived EVs as a cell-free therapeutic alternative have gained considerable interest. More than that, EVs can also be used as a vehicle to deliver bioactive factors. EVs derived from bone marrow mesenchymal stem cells (BM-MSCs) have been used to treat cartilage defects or osteoarthritis which are related to bone diseases [23,24], renal injury [25] or Graft Versus-Host Disease [26]. Small EVs derived from BM-MSCs are widely used in the treatment of myocardial infarction (MI). An important mechanism behind the regenerative potential of EV-MSCs is the immune-regulatory properties of these vesicles. In a recent paper, small EVs secreted by BM-MSCs were incorporated into alginate hydrogel to increase their retention in the heart, have been showed to reduce the apoptosis of cardiomyocytes and promoted the polarization of macrophages [27].

Among MSC-dervied EVs, ADSC-derived EVs stand out as novel mediators and biomarkers in the crosstalk between adipose tissue and other organs/tissues relevant in obesity and metabolic diseases. In recent years, EVs from ADSCs have attracted much attention for their role in metabolic dysfunction, in particular, obesity and its complications, but their role in diabetes and associated cardiovascular disease was very little discussed.

Diabetic nephropathy represents one of the most relevant chronic complications of diabetes and the major cause of end-stage renal failure [28]. At present, available clinical biomarkers including glomerular filtration rate (GFR), proteinuria and urinary sediment evaluation do not allow a specific diagnosis neither clarify disease staging. Therefore, the finding of non-invasive biomarkers could hinder the use of kidney biopsy, a procedure implying complication risks. Urine is an ideal source of biomarkers, particularly for diseases of the kidney and urinary tract, because it can be conveniently collected in large amounts without risk to the patient. Recent studies revealed that expression of urinary exosomal miRNA is changed in patients with type 2 diabetes mellitus (T2DM) [29,30]. Urinary exosomal miRNA content is altered in patients suffering from type 1 diabetes mellitus (T1DM) with incipient diabetic nephropathy and micro-albuminuria resulting in an up-regulation of miR-130a and miR-145 and a down-regulation of miR-155 and miR-424 [31].

Investigation of the pathophysiology of EVs and relevant mechanisms provides new opportunities in diagnosing and combatting metabolic disorders by the development of new therapeutic strategies in metabolic diseases such as diabetes and associated cardiovascular disease.

## 2. Adipose Tissue—Derived Stem Cells

### 2.1. Identification and Molecular Characteristics of Adipose Tissue Stem Cells

Isolation of cells from rodent adipose tissue was pioneered by Rodbell and collaborators in the 1960s. Later, in the 2001s, Zuk and collaborators described the isolation and cultivation of stem cells harvested from human lipoaspirate [32], and afterwards Bunnell and collaborators described the isolation of ADSCs from human adipose tissue. ADSCs are mesenchymal stem cells (MSCs) that are resident in large amounts in the adipose tissue and share key characteristics with bone marrow-MSCs, including the ability to adhere to plastic, to form fibroblastic like colonies (called CFU-F), and also their high proliferative capacity and remarkable capability to differentiate in vitro into mesenchymal lineages: osteoblasts, chondrocytes, adipocytes and skeletal myocytes [33,34]. It has been found that the SVF from adipose tissue provides 500 times more stem cells than bone marrow (only 0.01–0.001%), making ADSCs a reliable source of MSCs [35]. The number of isolated ADSCs can vary, depending on the isolation method. There are many processes for tissue digestion and cell isolation from adipose tissue, as those using collagenase, trypsin, dispase, or related enzymes.

Initially, a very specific panel of positive markers CD105, CD73 and CD90, and lack of CD45, CD34, CD14 or CD11b, CD79α or CD19, and HLA-DR surface molecules expression was stated to represent the prototypical ADSC hallmark [36]. Additionally, many other molecules have been reported in association with ADSCs, but their expression still remains controversial and is believed to be affected by donor-to-donor variability, differing protocols of isolation and expansion, and diverse experimental techniques. Until now there has been no definitive description of the immunophenotypic characteristics of ADSCs, and other markers were added to the previously reported ones. Of those, CD34, is a hematopoietic marker that has been reported both to be positive in SVF and lose its expression with time and culture passages of ADSCs [37], or to maintain its expression even at late culture passages [38]. Traktuev showed that a population of adipose-derived adherent CD34+ cells are resident pericytes that play an important role in vascular stabilization by reciprocal interaction with endothelial cells [39].

In the last few years, efforts have been made to identify and enrich adipose tissue progenitor cells, and Rodeheffer and collaborators distinguished two sub-populations in the adult murine SVF [40,41]. The two subpopulations of cells were separated by the non-immune cells based on both the lack of expression of CD45, CD31 and Ter119 markers (blood and endothelial cell markers) and the presence of CD29, CD34 and Sca-1/Ly6-A markers (mesenchymal and stem cell markers). The CD45^−^, CD31^−^, Ter119^−^, CD29^+^, CD34^+^, Sca-1^+^; CD24^+^ cells (adipocyte progenitor cells (APCs)) were capable of developing into functional WAT depots when they were transplanted into lipodystrophic mice, while the CD45^−^, CD31^−^, Ter119^−^, CD29^+^, CD34^+^, Sca-1^+^, CD24^−^ cells (preadipocytes), even being adipogenic in vitro, were not able to form WAT depots in vivo [41]. Thus, the CD24^+^ subfraction of CD34^+^Sca-1^+^ cells represent a stem cell-like population, whereas the CD24^−^ subfraction represents a pool of more committed APCs. Moreover, both populations of cells express platelet-derived growth factor receptor alpha (PDGFRα), a known marker of the adipocyte precursor cell lineage, and were highly adipogenic in vitro (Berry and Rodeheffer, 2013). Identification of new mice APC markers relevant for the in vivo behavior of APCs in WAT was accomplished by immunofluorescence localization, enlightening that at least a proportion of progenitor cells are located within the perivascular niche of capillaries and microvessels as pericytes (PDGFRα^+^/PDGFRβ^+^) [42,43]. Furthermore, it was observed that PDGFRα^+^ lineage contributes to all adipocytes in subcutaneous WAT [40] and expression of PDGFRα precedes PDGFRβ expression in almost all subcutaneous but only in a fraction of visceral ADSCs [44]. PDGFRβ^+^ expressing cells don’t usually differentiate but, when exposed to high fat diet, they turn into adipocytes in visceral WAT, and when exposed to cold, they form beige adipocytes in the subcutaneous depot.

In addition to the adipose precursors CD29, CD34, Sca-1 mentioned above, Wt1 (the protein encoded by the Wilms’ tumor 1 gene), known to function as a transcription factor and having post-transcriptional processing roles, is also important for adipocyte differentiation. Wt1 is highly expressed in mesothelial cells able to differentiate into adipocytes when exposed to adipogenic conditions in culture [45,46] and deletion of Wt1 in adult mice results in a considerable loss of fat tissue [47].

Recently, Prx1-Cre has been found to label subcutaneous and periaortic BAT and not visceral adipose tissue. Moreover, a portion of the Prx1-Cre labeled pericardial APCs express Wt1 [36,48]. Additionally, Myf5-Cre and Pax3-Cre are capable of tracing both white and brown depots [49,50], while PDGFRα-Cre labels both adipocyte progenitor cells and pre-adipocytes in subcutaneous and visceral depots. Therefore, these lineage tracing studies give valuable information on developmental origin of APCs from different adipose tissue depots. Going further, efforts have been made in order to identify subpopulations of adipose stromal cells in each of the fat depots, and the single cell RNA sequencing (scRNAseq) technology, recently developed as a powerful tool to deconvolve tissue heterogeneity came to help [51]. The results obtained with this technology are presented as an atlas of spatial clustering of subpopulation of cells based on log2 expression levels for select genes commonly used to purify adipose stem cells by fluorescent activated cell sorting (FACS) or lineage tracing experiments. Rondini and Granneman showed that more than 70% of non-immune stromal cells are represented by ADSCs that express genes for previously identified surface markers including PDGFRα, CD29 (Itgb1), CD34 (Cd34), and Sca-1 (Ly6a) [52]. There is a general consensus about the presence of two main populations of ADSCs (stated as ADSC1a and ADSC2), with an additional subpopulation (ADSC1b) present in inguinal WAT [53,54,55,56]. The relative ratio of total ADSC1 to ADSC2 in adult animals was found to be larger in epididymal WAT than in inguinal WAT. Functional assays suggest that the ADSC1 population expressed genes involved in lipid uptake and was more primed for adipogenesis, and ADSC2 expressed some genes that are associated with inflammation (prostaglandin synthesis), angiogenesis (endothelial cell migration), proteolysis (Pi16, Dpp4), as well as with immune signaling (Cd55) [54,55,56].

Conventionally, in humans, according to the International Society for Cellular Therapy (ISCT) criteria, mesenchymal stem cells are CD105, CD73 and CD90 positive cells, negative for HLA-DR, CD14, CD34, CD19 and CD45 [57], but other markers have been added later. A recent paper revealed that the following markers are attributed to ADSC phenotype: CD90, CD44, CD29, CD105, CD13, CD73, CD166, CD10, CD49e, and CD59 are positive markers, while CD31, CD45, CD14, CD11b, CD34, CD19, CD56, and CD146 are negative markers [58]. This panel of positive and negative markers on human ADSCs has been developed for in vitro propagated cells but it was observed that freshly isolated SVF contain numerous subpopulations, and to further isolate specific cell types, different combinations of markers have been chosen for FACS separation of cells.

### 2.2. Differentiation Potential of Adipose Tissue Stem Cells

The molecular mechanisms controlling the adipose tissue homeostasis have been intensively studied due to their potential application in the prevention and treatment of obesity and related disorders such as diabetes, dyslipidemia, and cardiovascular diseases. The expansion of adipose tissue as a response to metabolic challenges, either by hyperplasia or by hypertrophy, is strongly dependent on the adipogenic and immunomodulatory potentials of adipose-derived stem cells. Different studies have reported that ADSCs from obese subjects have decreased ability to differentiate into adipocytes in vitro, when compared with those from lean subjects [59,60,61]. A possible explanation is that the inflammatory cytokines in the adipose tissue from obese subjects impaired adipocyte differentiation [62]. Moreover, numerous studies showed that subcutaneous ADSCs have a higher adipogenic potential than those from visceral depots, both in humans [63,64] and in mice [65,66]. The ADSC differentiation capacity is also disrupted in patients with T2DM as indicated by global gene expression profiling revealed that ADSCs from T2DM donors have low levels of adipogenic genes compared with those from non-diabetic donors.

When exposed to specific growth conditions in vitro, ADSCs can be differentiated toward adipogenic [34], osteogenic [67], chondrogenic [68], myogenic [69], and neurogenic lineage [70].

Many transcription factors were shown to impact adipogenesis, some of them having a positive effect in terms of promoting adipogenesis, like C/EBPα, C/EBPβ, C/EBPδ, AP-1, E2Fs, PPARγ2, PRRX1, STAT5A, and others inhibiting adipogenesis, GATAs, PREF-1, Wnt-10b, and Wnt-5a, being involved in progenitor cell proliferation. Moreover, some cell cycle proteins such as cyclin D, p21, p27^KIP^ (cyclin-dependent kinase inhibitors), and estrogen, insulin, IGF-1 (hormones) are involved in adipocyte development [71,72,73]. C/EBP-β and C/EBP-δ are the first transcription factors induced during adipogenesis and play a crucial role in directing the differentiation process. While C/EBP-β synthesis decays, C/EBP-α and PPARγ2 transcription is continuously stimulated by C/EBP-α binding to CEBPs regulatory elements. The PPARγ2 transcription is controlled also by transcription factor SREBP1 and KLF family [74,75]. It has been found that PPARγ can promote adipogenesis in C/EBP-α -deficient cells [76], but C/EBP-α does not function similarly in the absence of PPARγ2 [77]. In the last phase of adipogenic differentiation, genes associated with mature adipocytes such as fatty acid synthase, glycerophosphate dehydrogenase, acetyl CoA carboxylase, glucose transporter type 4, insulin receptor, adipocyte-selective fatty acid binding protein (aP2) are markedly expressed, and numerous lipid droplets are formed [78].

Different studies demonstrated that factors and pathways that stimulate osteogenesis inhibit adipogenesis. Conversely, adipogenic induction inhibits osteogenesis [79]. The balance between adipogenesis and osteogenesis is important to keep homeostasis in the organism. Osteogenic differentiation of ADSCs in vitro can be influenced by different factors: methods used for isolation, cell culture medium used for differentiation, and agents used for enhancing osteogenic differentiation ability, FGF-1, BMP-2, BMP7 or BMP9 [80,81,82]. In vitro, osteogenic differentiation is usually induced using DMEM cell culture media supplemented with 10% of fetal bovine serum and a mixture of dexamethasone, ascorbic acid and β-glycerophosphate for a period of 21 days. Dexamethasone induces the expression of Runx2 mediated by β-catenin activation, ascorbic acid acts as a cofactor for proline- and lysine-hydroxylases that have a role in collagen synthesis, and β-glycerophosphate serves as a phosphate source for bone mineralization and induces osteogenic genes. Signaling pathways involved in osteoblast differentiation are that of ERK2 that activates Runx2, JNK/MAPK that is involved in downstream transduction of signals from BMP-2 [83], AMPK activated by adiponectin [75] and a very important role has been attributed to TGF-β/SMAD pathway [84], whose activation leads to increased extracellular calcium deposition, increased alkaline phosphatase (ALP) production and increased expression of osteogenic genes as Runx2, ALP and osteocalcin [85]. In addition, some exosomal microRNAs have been proved to have a role in bone differentiation, acting as regulators of the balance between adipogenic and osteogenic differentiation in human ADSCs.

The process of chondrogenic differentiation of MSCs in vitro is usually performed with pellet or aggregate culture system, with DMEM high glucose media supplemented with FBS and antibiotics and addition of factors as TGFβ, BMP and/or IGF (reviewed by Boeuf and Richter [86]; Somoza et al., [87]). Chondrocytes generated from MSCs expressed classical genes/proteins as native chondrocytes, e.g., type II collagen and aggrecan. However, it is also possible to identify hypertrophy-associated genes, as type X collagen, ALP and MMPs [87]. The data analysis also demonstrated that MSCs during chondrogenesis express classical markers of hyaline cartilage as aggrecan, SOX9, COL2 and others, but also express COL10, Runx2, ALPL, and MMP13 which are present in hypertrophic cartilage.

Subcutaneous ADSCs can differentiate into endothelial cells in vitro. Different subpopulations of cells from subcutaneous SVF, CD13^+^CD34^+^ [88], CD34^+^CD31^−^, Flk-1^+^ [89], and CD31^−^CD34^−^c-kit^−^ [88], have been shown to form vessel-like structures by applying matrigel-plug assay subcutaneously in mice. Two studies confirmed that phosphatidylinositol 3-kinase (PI3K) is a key signalling pathway in the process of ADSC differentiation toward endothelial cells [90,91].

ADSCs could also differentiate into smooth muscle cells [92,93], with TGF-β1/Smad pathway activation having an important role [94].

ADSCs can differentiate to cardiomyocytes as well. Studies done by Planat-Bénard and collaborators revealed that ADSCs can be differentiated into cardiomyocyte-like cells [95] and, when these cells were injected in rats with a model of chronic myocardial infarction [96] and in nonhuman primates [97], they significantly improved the heart function.

Not only subcutaneous ADSCs have beneficial effect on improving vascular and cardiac function, Madonna and collaborators described that visceral (periepididymal and omental) ADSCs isolated from mice, cultured in methylcellulose medium, could spontaneously undergo neovascularization differentiation, forming CD31^+^CD34^+^ tube-like structures [98]. Other studies have shown that KDR^+^CD34^−^CD31^−^ ADSCs from human visceral adipose tissue or from murine visceral depot, intravenously or intramuscularly injected in mice with femoral artery ligation, enhanced capillary density and Doppler tissue perfusion scores [90,99].

ADSCs isolated from cardiac tissue were prone to differentiate more into cardiovascular cells as compared with ADSCs from other sources [100]. On the other hand there are studies that have shown that epicardial ADSCs have higher cardiomyogenic potential than pericardial ADSCs [101], the latter having a better cardiac reparative activity than subcutaneous ADSCs, being able to differentiate toward myogenic lineage and supporting vasculogenesis. The cardioprotective effects of epicardial ADSCs have not been reproduced in a genetic model of obese hyperlipidemic and type-2 diabetic rat.

## 3. Extracellular Vesicles from Adipose Tissue Stem Cells

### 3.1. Classification and Molecular Properties of Adipose Tissue Extracellular Vesicles

There is no consensus on specific markers of EV subtypes, such as endosomal origin exosomes (small EVs) and plasma membrane-derived ectosomes (microparticles/large EVs), therefore assigning EVs to a particular biogenesis pathway remains very difficult. Due to an increased interest in the EVs field and of higher number of published papers working with these EV subtypes, whose size and amount often make them difficult to obtain as relatively pure preparations, and to characterize properly, an improved guideline have been published in order to help scientists to make strong conclusions on the involvement of specific populations of EVs in physiological or pathological condition. A list of minimal information for studies of EVs (MISEV2018) was provided, covering EV separation/isolation, characterization, and functional studies. This updated ISEV statement reflects an improved understanding in EV biology, which has resulted in a consensus to promote meaningful changes to nomenclature and experimental approach. MISEV2018 guidelines advise the authors to use operational terms for EV subtypes that refer to (a) physical characteristics of EVs, such as size (small EVs (sEVs) and medium/large EVs (m/lEVs), with ranges defined, for instance, respectively, <100 nm or <200 nm [small], or >200 nm [large and/or medium]) or density (low, middle, high, with each range defined); (b) biochemical composition (CD63^+^/CD81^+^-EVs, Annexin A5-stained EVs, etc.); or (c) descriptions of conditions or cell of origin (podocyte EVs, hypoxic EVs, large oncosomes, apoptotic bodies) [102].

Considering the fat type and location, adipose tissue-derived EVs can be divided into: EVs secreted from subcutaneous or visceral fat and EVs secreted from WAT or BAT.

Molecular properties of adipose tissue EVs rest on their composition in lipids, proteins, and nucleic acids. Basically, the biomolecular content of EVs is similar to that of the source cell, any differences in adipose tissue-derived EVs composition resting on the content variability of the original fat cell type.

Adipose tissue EV release has been widely studied in explants from both adipose visceral and subcutaneous tissues [103,104], as well as in in vitro differentiated adipocytes and in adipose tissue stem cells (ADSCs) [103]. However, most of the in vitro studies used the murine 3T3-L1 pre-adipocyte cell line differentiated to mature adipocytes and only a few used human adipocytes and adipose tissue extracts [105].

Analyses of microRNA (miRNA) profiles, showed that adipocyte-derived small EVs exhibit abundant miRNA content, many of which are up-regulated, such as miR-103, miR-146b, miR-148a [106,107,108]. Considering the length of adipogenesis induction, 3T3-L1 small EVs, registered a time dependent increment of adipogenesis-related gene transcripts expression, peroxisome proliferator-activated receptor γ2 (PPARγ2), adiponectin and leptin [109]. Therefore, EVs from adipogenic induced 3T3-L1 cells, doubled adiponectin content, while fatty acid-binding protein 4 (FABP-4) and preadipocyte factor 1 (PREF-1) levels decreased [105]. Adiponectin, as well as a small amount of resistin were found also in small EVs from serum [110]. After adipogenesis, the lipid composition of EVs was also enriched in phosphatidiyl serine and arahidonic acid and in long chain omega-3 fatty acid decosahexaenoic acid [111]. Protein marker expression of CD9, CD36, TSG101 and Alix remained unchanged, after 3T3-L1 cell differentiation [105]. In normoxic and hypoxic conditions, 231 proteins were identified in 3T3-L1 small EVs, including enzymes involved in “de novo” lipogenesis, mostly when the hypoxic environment induced by adipocyte hypertrophy was mimicked [112]. Moreover, besides adipocyte specific proteins, several immunomodulatory proteins, such as tumor necrosis alpha (TNF-α), macrophage-colony-stimulating factor (MCSF) and retinol binding protein4 (RBP-4), have been reported within the small EVs [103].

Human adipose tissue isolated ADSCs release small EVs that have been shown to contain small RNA species like miRNAs, small nucleolar RNAs (snoRNAs) and mostly transfer RNAs (tRNAs) [113]. Protein secretory profile of ADSCs is considered almost specific to each person, thus forming a heterogeneous population of cells that may produce equivalent EVs [114].

Differentiated human adipocytes-derived EV content is characterized by adipose specific markers FABP-4 as well as adiponectin and by a number of inflammatory adipokines, including macrophage migration inhibitory factor (MIF), TNFα, MCSF, and RBP-4 [103]. Regarding the adipokine profile, visceral adipose tissue EVs have a significantly higher concentration of interleukin -6 (IL-6), MIF, and monocyte chemoattractant protein-1 (MCP-1) compared to those from subcutaneous adipose tissue. The content of EVs produced by both subcutaneous and visceral adipose tissue is also rich in adiponectin. In contrast to adiponectin-negative EVs produced mainly by stromal cells, adiponectin-positive EVs are produced exclusively by adipocytes.

Since the release of small EVs by BAT is increased after cAMP treatment, down regulation of specific marker miR-92a was also observed in both murine and human small EVs after cold exposure-dependent cAMP activation (Chen et al., 2016).

### 3.2. Physiological Functions of Adipose Tissue Extracellular Vesicles

Adipocyte released EVs influence the vascular health of the adipose tissue, being important means of vascular homeostasis regulation by neovascularization and angiogenesis [105], ADSC small EVs promoting vascular endothelial cell migration and proliferation and stimulating neo-vessel formation [115,116].

Adipocyte-derived EVs (including large EVs and small EVs) may function as adipokines contributing to adipose tissue homeostasis or dysfunction. An important role of EVs isolated from adipose tissue is their capacity to mediate the endocrine connection between maternal adipose tissue and fetal growth, being unfortunately also responsible for fetal overgrowth [117]. Since adipocyte-derived EVs contain large amount of adiponectin, a crucial adipokine for glucose and lipid metabolism, also involved in fatty acid oxidation and insulin sensitivity [118], small EVs through their rich adiponectin content may be involved in distant cell metabolism.

Adipocyte derived EVs have also an important role in paracrine regulation of adipocyte metabolism [105]. Microvesicles and exosomes released by adipocytes containing glycosylphosphatidylinositol (GPI)-anchored proteins, CD73 and Gcel have role in esterification and in lipolysis inhibition [119] the small EVs may be effective players in adipocyte intercommunication [120].

The paracrine cross talk between adipocytes and macrophages is also regulated by adipose tissue-derived EVs. When primary monocytes differentiate into macrophages, the most effective EVs were adiponectin-positive and visceral ones compared to adiponectin-negative and subcutaneous EVs [103]. Moreover, the exposure of hepatocytes to subcutaneous and visceral adipose tissue-derived EVs led to a negative association between Akt signaling and glucose-6-phosphatase gene expression [103]. There is evidence of adipocyte-derived EV communication with the immune cells as well as their influence on whole body. EVs are implicated in regulation of hepatic insulin signalling and immunity as shown in vitro by their ability to reduce the proliferation rate of stimulated T lymphocytes and by controlling monocyte to macrophage differentiation.

## 4. Pathobiological Significance of Extracellular Vesicles from Adipose Tissue Stem Cells in Diabetes and Associated Cardiovascular Disease

### 4.1. Extracellular Vesicles from Adipose Tissue Stem Cells as Diagnostic Biomarkers

Biomarkers have a notable role in the prediction, diagnosis, and assessment of the therapeutic success in common multifactorial metabolic diseases, such as T2DM and obesity. Numerous studies have revealed that EVs are significant tools for biomarker discovery in early detection, due to their content that can be transferred, protected from degradation, from organs, tissues, and cells into body fluids. For instance, EVs released into the plasma and urine are considered specific biomarkers for the pathogenesis of T2DM [121].

The adipose tissue, among other sources as pancreatic islets (particularly β-cells), liver, skeletal muscle, vascular endothelial cells, and tissue and plasma macrophages are recognized candidates for relevant EVs.

Adipocyte hypertrophy and hyperplasia are both involved in the significant increase in adipose tissue mass in obese and T2DM patients [121]. Adipocyte hypertrophy has also been associated with the elevated production and release of EVs, characterized by the expression of perilipin A [122,123]. Perilipin A was identify to be a plausible biomarker of circulating EVs originating from the adipose tissue and a feasible target for novel diagnostics development for assessment of obesity-related adipocyte dysfunction and metabolic complications [122,123]. Furthermore, it has been shown that in obesity, plasma EVs contain high amount of cystatin C and CD14, that have been correlated with high risk for myocardial infarction, vascular disease mortality and subsequent vascular events, in patients with vascular diseases [124]. The expression of cystatin C in plasma EVs was positively associated with reduced systemic inflammation, diminished HDL-cholesterol levels and metabolic syndrome, while expression of CD14 was negatively related to adipose tissue abundance, dyslipidaemia and decreased risk for the T2DM development [125]. Interestingly, the generation of these two proteins was associated with development of cardiovascular complications. Cystatin C and CD14 expression was reported to be pronounced in adipose tissue, suggesting a potential contribution of the adipose tissue-derived EVs to the advance of metabolic complications of obesity [126]. Moreover, in obesity, adipose tissue develops resistance to insulin-mediated suppression of lipolysis which enhances the release of small EVs enriched in adipose fatty acid binding protein (aP2), leading to amplified liver glucose output and diabetes [127]. Interestingly, it has been indicated that ADSCs from the subcutaneous depots of obese subjects have lower expression levels of stem cell markers, such as octamer-binding transcription factor 4 (Oct4), Sall4, SRY-box transcription factor 15 (Sox15), Kruppel-like factor 4 (KLF4), and they also have a higher expression of the stem cell marker B cell-specific moloney murine leukemia virus integration site 1 (Bmi-1), compared with ADSCs derived from subcutaneous depots of lean donors [128]. In contrast, the same study showed that ADSCs derived from omental depots of obese subjects have greater expressions of Oct4, Sall4, Sox15, KLF4 and BMI1 compared to omental depots of the lean donors. In obese patients, the removal of large volume of adipose tissue by liposuction improves general heath and reduces leptin, increases adiponectin plasma levels and improves the inflammatory status [129] as result of decreasing the whole body fat mass containing both pathological adipocytes and inflammatory EVs from adipocytes and also ADSCs. On the other hand, liposuction-subjected normal weight patients show no significant changes or slight improvements in these metabolic parameters due to the removal of only small quantities of adipose tissue [130]. These findings suggest that obesity changes the adipose stem cell niche and its EVs output and is dependent on the depot-specific source of the adipose tissue stem cells.

**Figure 1:**
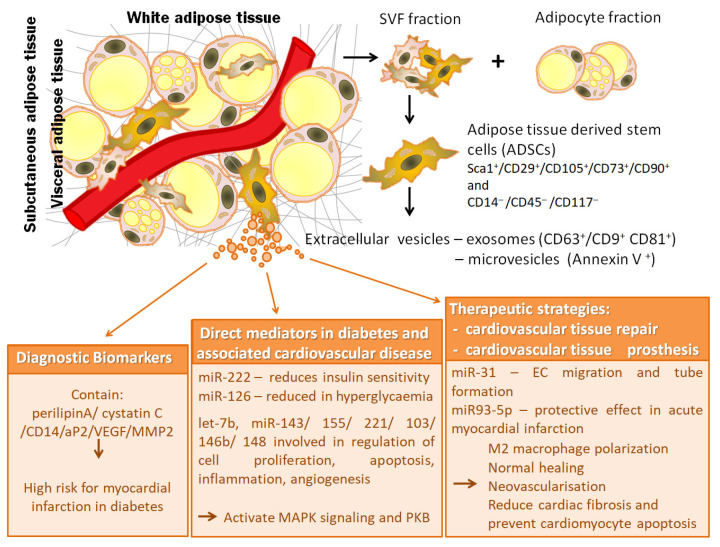
Relevance of extracellular vesicles from adipose tissue stem cells in pathophysiology of diabetes and associated cardiovascular disease: extracellular vesicles as diagnostic biomarkers, direct mediators and therapeutic strategies.

Besides, it was shown that EVs released from cultured 3T3-L1 adipocytes contain about 140 miRNAs specific to the adipocytes. These adipocyte-derived EVs also harbor mRNAs coding for adiponectin, resistin and PPARγ, that can be transported into cultured macrophages inducing angiogenesis [121]. In obesity, elevated levels of miR-222, a negative regulator of insulin sensitivity in adipocytes, were found both in adipose tissue and in circulating small EVs [131]. In pre-adipocytes from obese subjects, several miRNAs were modified compared with lean subjects, miR-221, miR-125b, miR-34a and miR-100 were up-regulated while miR-130b, miR210 and miR-185 were down-regulated [132]. Thus, miRNAs associated with EVs derived from adipocytes, like those derived from vascular cells and macrophages, seem to reflect the functional state of the adipose tissue and may be useful for following the development of obesity and may be considered markers for diagnostic. See Figure 1 for a summary of the proposed markers of disease, disease mediating molecules and therapeutic targets found in ADSC-derived EVs.

The study of biomarkers from adipose tissue-derived EVs for cardiovascular diseases is still in its beginning and current available data from human clinical trials showing promising candidate marker molecules on EVs are summarized in Table 1 below. The EV-based biomarkers could provide essential diagnostic and prognostic values for better treatment of patients.

Currently, no definitive marker of adipocyte-derived EVs has yet been decided and no consensus was established regarding the characteristic EV cargo. This makes it difficult to interpret findings on circulating EVs in obesity and metabolic diseases as well as in type 2 diabetes [133]. The miRNAs carried by EVs secreted by adipose tissue could provide new diagnostics biomarkers to distinguish metabolically healthy versus metabolically unhealthy obesity, and new approaches to deliver miRNAs that target genes in the liver and other tissues for metabolic syndrome management.

### 4.2. Pathological Roles and Responsible Mechanisms of Adipose Tissue Extracellular Vesicles

There is growing evidence that adipose tissue is a major contributor of circulating miRNAs and that miRNAs carried by adipocytes-derived EVs, may possess hormone-like functions, communicating with other tissues to coordinate metabolic homeostasis and energy balance [131]. When these systems are disturbed, they may also participate to the pathophysiology of metabolic diseases, such as obesity, lipodystrophy, type 2 diabetes, and metabolic syndrome, and they may also contribute to insulin resistance in these diseases [131].

Despite the fact that there are many reports regarding the importance of adipose tissue-derived EVs in development of metabolic diseases, the role of EVs from ADSCs in obesity and related pathological conditions remains unclear.

Some studies have shown that ADSC-derived EVs might increase insulin sensitivity through their ability to decrease inflammation [134]. Recently, it was demonstrated that ADSC-derived large EVs from normal subjects are rich in miRNAs, including two members of the let-7 family, with role in angiogenesis [135]. These ADSC-derived large EVs can promote the migration and invasion abilities of endothelial cells, suggesting their pro-angiogenetic potential. These data are in agreement with a previous study indicating that miR-31 carried by ADSC-derived large EVs contributes to the migration and tube formation of endothelial cells by targeting and suppressing factor-inhibiting hypoxia-inducible factor-1 Also, it has been reported that adipocyte-derived large EVs carry a subset of miRNAs, such as let-7b, miR-143, miR-155, and miR-221 that are involved in the control of cell proliferation, apoptosis, inflammation and angiogenesis in adipose and vascular tissues [109]. Other miRNAs contained by adipocyte-derived large EVs are miR-103 and miR-146b/148. Moreover, it was reported that circulating adipose-derived small EVs transport miR-99b, that is involved in regulation of liver fibroblast growth factor 21 (FGF21), contributing in this way to the control of metabolic homeostasis, as well as to systemic insulin resistance [136]. These data suggest the positive role of healthy ADSC-derived EVs on the metabolic homeostasis.

In contrary, there are data that indicate potential pathogenic role for ADSC-derived EVs which act at distance in various metabolic diseases. In this context, studies have shown that EVs from obese ADSCs, either from subcutaneous or from visceral depots, contain reduced levels of vascular endothelial growth factor (VEGF) and matrix metalloproteinase-2 (MMP-2), compared to those derived from the same compartments in non-obese individuals, suggesting that they may have lowered pro-angiogenic potential [133]. Moreover, it has been noticed that small EVs secreted by obese ADSCs contain impaired levels of miR-126, which are damaged by the additionally hyperglycaemic milieu [137].

In metabolic syndrome, it has been demonstrated that EVs derived from porcine ADSCs contain high levels of some miRNAs, including miR-146a-3p, miR-30c-1-3p, miR-7, miR-148a-5p, miR-374a-3p, miR-23a, miR-132, miR-129b [138]. These miRNAs target transcription factors, as well as genes, implicated in the development of metabolic syndrome and its complications, including validated transcriptional targets of AP1 family members Fra1 and Fra2, Class A/1 (Rhodopsin-like receptors), and Peptide ligand-binding receptors [138]. Additionally, the same authors found that, in metabolic syndrome, porcine ADSCs contain seven upregulated miRNAs, including miR-196a, miR-301, miR-27b, miR-7a, miR-7c, and miR-7e, miR-425 and three downregulated miRNAs, miR-99a, miR-708, and miR-148a, which regulate 35 senescence-associated genes, particularly involved in MAPK signaling, suggesting that these miRNAs may modulate ADSC senescence [139]. The evaluation of these miRNAs in ADSCs-derived EVs could be useful in following the ADSC senescence in metabolic diseases. Interestingly, it has been reported that small EVs derived from ADSCs from omental depots of obese patients are enriched in lincRNA-VLDLR, and also have a higher expression of metastasis-associated lung adenocarcinoma transcript 1 (MALAT1), compared to small derived from ADSCs of omental depots of lean donor [128]. These specific long noncoding RNAs (lncRNAs) can be transferred to recipient cells to regulate gene expression. Thus, it was found that internalization of ADSC-derived small EVs by human dermal fibroblasts induced increased expression of MALAT1, which is involved in the modulation of several molecular signaling pathways and affects proliferation, cell cycle, migration, and angiogenesis [140,141]. Some evidence shows that ADSC-derived small EVs activate the protein kinase B (PKB, also known as Akt) and extracellular signal-regulated kinase (ERK) signaling pathways, which are involved in the proliferation, migration, and tube formation of endothelial cells. Moreover, it have been demonstrated that ADSC-derived small EVs play an inhibitory role in cell apoptosis by transferring and regulating proteins and miRNAs [142].

Consequently, although the data presented above suggests that the profiles of EVs derived from adipose tissue change in obesity and metabolic disease, it is not yet clear whether EVs could be used only as markers of disease or if they also play an important functional role in inter-tissue communication.

## 5. Adipose Tissue Stem Cell—Derived Extracellular Vesicle Based Therapeutics for Diabetes and Associated Cardiovascular Disease

### 5.1. Effects of Adipose Tissue Extracellular Vesicles in Cardiovascular Tissue Repair

Nonalcoholic fatty liver disease (NAFLD), and especially NAFLD associated with liver fibrosis, is a known risk factor for cardiovascular disease [143,144]. The EVs shed from visceral ADSCs of obese subjects integrated in cultured hepatocytes and hepatic stellate cells were shown to dysregulate the transforming growth factor β (TGFβ) signaling pathway compared to EVs from lean subjects, leading in obesity-associated T2DM to expression profiles similar to those in NAFLD [104]. However, EVs derived from MSCs were shown to alleviate tetrachloride induced liver fibrosis in a mouse model [145] and, in a similar model, ADSC-derived EVs enriched with miR-181-5p reduced liver fibrosis even more efficiently compared to native ADSC-derived EVs, by inhibiting the STAT3/Bcl-2/Beclin 1 pathway [146]. Another study suggests that EVs from adipose tissue can contribute to systemic insulin resistance with effects on both hepatocytes and skeletal muscle cells. In hepatocytes, the studied EVs inhibited insulin-induced Akt phosphorylation, while in myotubes they exerted either an insulin inhibiting or stimulating effect, depending on the subject from which EVs were obtained. This study also found that the number of subcutaneous EVs was reduced in obesity and that omental EVs can predict liver dysfunction as indicated by elevated liver enzymes [147]. As insulin resistance is a known risk factor for cardiovascular disease [148], EVs from ADSC might play roles in both the pathogenesis and prevention of adverse cardiovascular outcomes. Supporting this claim, a high-fat diet mouse model, intraperitoneally administered ADSC-derived small EVs led to improvements in glucose tolerance and insulin sensitivity, decreased the serum levels of triglycerides and total cholesterol and alleviated the hepatic steatosis observed in the control group [134].

The ADSC-derived EVs play roles not only in alleviating known risk factors of cardiovascular disease, but it also has been shown that they could be useful for therapeutic approach of acute cardiovascular events. In vitro, ADSC-derived EVs prevented cardiomyocyte apoptosis induced by oxidative stress, and in vivo animal models of cardiac infarction, intravenous administrated ADSC-derived EVs improved echocardiographic and hemodynamic outcomes by leading M2 macrophage polarization and immune cell trafficking, as shown by the activation of the S1P/SK1/S1PR1 pathway [149], and reduced the cardiac fibrosis and pro-inflammatory cytokine levels while promoting angiogenesis, with miR-126-enriched EVs having a larger effect [150]. Additionally, in a different study, miR-93-5p-entiched EVs showed an even greater protective effect on acute myocardial infarction-induced myocardial damage than unmodified ADSC derived EVs, by reducing Atg7-mediated autophagy and suppressing TLR4 [151].

The angiogenesis-inducing activity of ADSC-derived EVs was also shown to serve therapeutic functions in the wound healing of healthy or diabetic animals [152,153], this effects being at least partly mediated by PI3K/Akt activation in fibroblasts leading to increased proliferation, migration, and collagen synthesis [154]. Moreover, EVs released after stimulation with hydrogen peroxide (H_2_O_2_) of ADSCs seem to promote neovascularization and inflammation and reduce apoptosis after ischemic-reperfusion injury [155] pointing to the importance of the cell culturing conditions.

ADSCs intravenously administrated to atherosclerotic rats lowered VEGF, ET-1, VCAM-1, and ICAM-1 markers of endothelial activation, CRP, IL-6, and TNFα inflammation markers, improved the lipidic profile and reduced the size of atherosclerotic plaques [156]. However, the observed improvements are likely a cumulative of ADSC differentiation and subsequent tissue repair and paracrine secretion of EVs and other soluble factors. An in vitro study aiming to distinguish ADSC-derived EV effects from differentiation of ADSCs to endothelial cells showed that EVs alone are able to decrease the apoptosis of endothelial cells, presumably by the reduction of miR-324-5p expression, a putative marker and effector of atherosclerosis [157].

**Figure 2:**
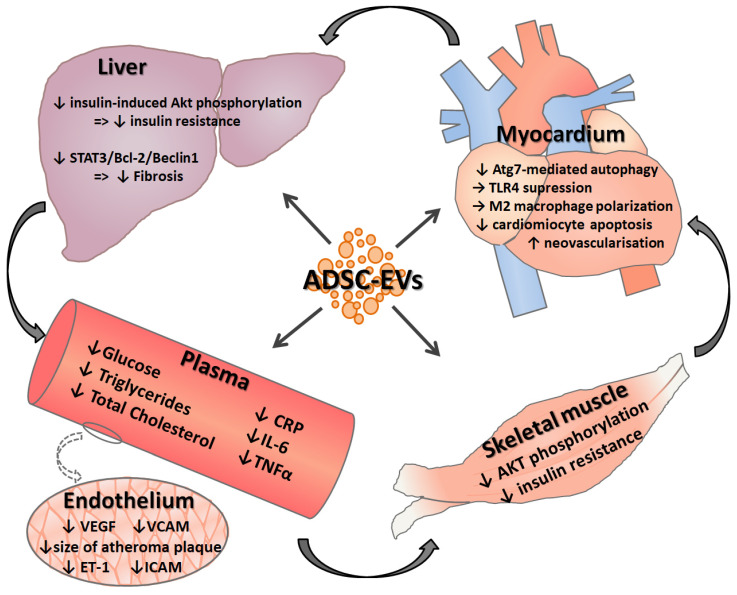
Effects of extracellular vesicles from adipose tissue stem cells on different organs involved in the pathology of diabetes and associated cardiovascular disease.

Figure 2 summarizes the reported actions of ADSC-derived EVs in different organs and body compartments.

### 5.2. Potential Use of Adipose Tissue-Derived Stem Cells and Extracellular Vesicles Generated by Them in Cardiovascular Tissue Prostheses

ADSCs could be used in the production of vessel prosthesis due to their ability of differentiating in both endothelial as well as smooth muscle cells. For this purpose, multiple stimuli are to be employed: VEGF and physiological shear stress are enough to differentiate human ADSCs seeded on collagen or acellular valve matrices into endothelial cells [158], while for the differentiation in smooth muscle cells, uniaxial mechanical strain at a frequency of 1 Hz, as well as TGF-β1 stimulation were successfully employed [159]. A similar approach was used to produce from a polyglycolic acid mesh and ADSCs a small diameter elastic blood vessel wall with contractile function [160]. Moreover, ADSCs incorporated in bioprinted scaffolds presented good viability, proliferation and differentiation in both endothelial cells and smooth muscle cells [161]. Different materials were used for the scaffold, such as polycaprolactone [162] and in the case of synthetic scaffolds use, additional collagen I, III or fibronectin coating helps in ADSCs retention [163].

It was also noted that co-incubation of smooth muscle cells with ADSCs pre-differentiated towards smooth muscle cells presents better functional traits compared to smooth muscle cells monoculture due to the initial paracrine function of ADSCs and their latter differentiation into fully functional smooth muscle cells. In experiments using static repopulation of acellular valves, ADSCs migrated only superficially [164], highlighting the need of mechanical stimulation for ADSCs migration and differentiation.

Cell therapy using ADSCs is not only sought for the differentiation capabilities of these cells but also for their paracrine function with growth hormones, cytokines and small EVs as secreted factors. As a way to retain ADSCs at their intended site of action for them to exert their paracrine actions, ADSCs-carrier sheets were developed and showed promising results in the treatment of myocardial infarction such as increased survival post myocardial infarction [165,166,167] in animal models.

ADSCs incorporation in macroencapsulation devices was also used to raise the biocompatibility of the biomaterials employed—polytetrafluoroethylene and polyethylene terephthalate—by modulating the immune responses, reducing fibrosis and promoting angiogenesis. This effect is due to the paracrine function of ADSCs as the cells themselves were restricted to the inside of the device [168].

## 6. Conclusions and Perspectives

There are numerous studies on EVs secreted from ADSCs, but few of these are original research that has investigated their involvement in diabetes and associated cardiovascular disease. It is well known that, ADSC-derived EVs seem to have potential roles in different physiological and pathophysiological situations, both as a direct effector to promote or prevent pathogenesis and as a delivery system to target miRNAs to cells. Besides their potential as markers for early diagnosis of the disease, ADSC-derived EVs were found to exert effects on angiogenesis, cell survival and apoptosis, inflammation, tissue regeneration, and reduction of disease pathology.

This review offers a systematic presentation on EVs secreted from ADSCs and their pathobiological significance in diabetes and associated cardiovascular disease. Additionally, responsible mechanisms and their determined roles on cardiovascular tissue repair and prostheses are discussed. It has thus been shown that adipose tissue is a real source of stem cells, and EVs produced by ADSCs appear to have potential roles in diabetes and cardiovascular pathology, both as biomarkers and direct contributors as well as a therapeutic option.

Future investigations are required to identify the ADSC-derived EV signature, causally involved in the pathogenesis of metabolic disorders, such as diabetes and associated cardiovascular disease, and those that reflect the temporal and individual disease states.

Although the results of the studies show that EV profile changes in diabetes and cardiovascular disorders, it cannot be stated with certainty that EVs are only a marker of the disease or if they actively participate in the progression of pathological processes or, on the contrary, in their regression.

Given ADSC-derived EVs potential for nanoparticle-based disease therapy, future research should consider these therapeutic abilities especially in pathology of cardiovascular diseases in diabetes. However, much more research is needed that pilot studies on patients be considered.

## Figures and Tables

**Table 1:** Clinical studies on ADSC-derived EVs and adipocyte-derived EVs as markers in cardiovascular disease.

Condition/ Disease	Patients	Extracellular Vesicles	Main Findings	Study
Obesity	- 14 obese patients, w/o diabetes, not on medication for metabolic syndrome alleviation, vs. 23 lean control subjects	plasma EVs	- EVs are increased in the circulation of obese subjects - circulating EVs numbers correlate with insulin resistance - perilipin A is a biomarker for circulating EVs from stressed adipocytes.	Eguchi A, et al., 2016 [123]
Cardio- vascular diseases	- 1060 patients from a prospective study cohort	plasma medium/ large EVs	- Cystatin C, Serpin F2, and CD14 levels in plasma medium/large EVs are correlated with MI incidence, vascular mortality and all-cause mortality. - CD14 levels were further correlated with the occurrence of ischemic stroke.	Kanhai DA, et al., 2013 [124]
Obesity and Cardio- vascular diseases	- 1012 patients with clinically manifest vascular disease	plasma EVs	- Cystatin C levels are positively related to metabolic complications of obesity - CD14 are negatively related to metabolic complications of obesity.	Kranendonk ME, et al., 2014 [125]
Obesity and diabetes	- 6 non-obese subjects - 9 obese with T2DM subjects - 13 obese without T2DM subjects	pre-adipocytes from sub- cutaneous fat and, by extension, EVs from pre- adipocytes	- miR-221, miR-125b, miR-34a and miR-100 were up-regulated in obese subjects - miR-130b, miR210 and miR-185 were down-regulated in obese subjects	Ortega FJ, et al., 2010 [132]

## Abbreviations

ADSCs	Adipose tissue-derived stem cells
EVs	extracellular vesicles
WAT	white adipose tissue
BAT	brown adipose tissue
SVF	stromal vascular fraction
MSCs	mesenchymal stem cells
PDGFRα	platelet-derived growth factor receptor alpha
PDGFRβ	platelet-derived growth factor receptor beta
Wt1	Wilms’ tumor 1
scRNAseq	single cell RNA sequencing
FACS	fluorescent activated cell sorting
ISCT	International Society for Cellular Therapy
PI3K	phosphatidylinositol 3-kinase
miRNA	microRNA
PPARγ2	peroxisome proliferator-activated receptor γ2
TNF-α	tumor necrosis alpha
MCSF	macrophage-colony-stimulating factor
RBP-4	retinol binding protein4
snoRNAs	small nucleolar RNAs
tRNAs	transfer RNAs
MIF	macrophage migration inhibitory factor
IL-6	interleukin -6
MCP-1	chemoattractant protein-1
FGF21	fibroblast growth factor 21
VEGF	vascular endothelial growth factor
MMP-2	matrix metalloproteinase-2
lncRNAs	long noncoding RNAs
PKB	protein kinase B
ERK	extracellular signal-regulated kinase
NAFLD	nonalcoholic fatty liver disease
TGFβ	transforming growth factor β
T2DM	type 2 diabetes
MISEV	minimal information for studies of extracellular vesicles
